# Synthesis and Degradation Properties of Sericin/PVA Hydrogels

**DOI:** 10.3390/gels9020076

**Published:** 2023-01-17

**Authors:** William Ekasurya, Joses Sebastian, Dita Puspitasari, Putri P. P. Asri, Lia A. T. W. Asri

**Affiliations:** Materials Science and Engineering Research Group, Faculty of Mechanical and Aerospace Engineering, Institut Teknologi Bandung, Jalan Ganesa 10, Bandung 40132, Indonesia

**Keywords:** sericin, hydrogel, PVA, wound dressing, diabetic foot ulcer

## Abstract

One method of treating diabetic foot ulcers, mainly superficial and deep ulcers, is using a wound dressing in the form of a hydrogel. Sericin derived from silkworm cocoons is a promising hydrogel material candidate because it has anti-inflammatory properties and stimulates collagen production. Sericin was combined with PVA to increase the stability of the resulting hydrogel. Sericin/PVA hydrogel was prepared using the freeze–thawing method with variations in the solution concentration and volume ratio of PVA and sericin. Sericin was successfully extracted using an autoclave method. The FTIR results confirmed that sericin extracted from the cocoon had a dominant secondary structure in the form of a β-sheet. Hydrogel with a concentration of 4% with a 1:1 ratio of PVA and sericin showed good stability and resulted in a hydrogel with characteristics that combine PVA and sericin. The resulting hydrogel had an average pore size range of 24–191 µm and a porosity range of 70–85%, which meets the requirements for wound dressings. Through degradation testing in PBS solution, it was found that the sericin/PVA hydrogel experienced degradation of 60–75% after 672 h of testing.

## 1. Introduction

A diabetic foot ulcer is a condition that can affect people with type 1 and type 2 diabetes mellitus and is triggered by minor trauma. Every year there are 9.1 to 26.1 million people in the world who experience these ulcers. Meanwhile, 15 to 25% of patients with diabetes mellitus will develop diabetic ulcers in their lifetime [1]. One treatment that can be completed for stage 1 and 2 diabetic foot ulcers is the use of a wound dressing expected to relieve symptoms, provide protection against wounds, and promote healing [2]. Selection of the right wound dressing should be based on its ability to maintain a moist environment, promote epidermal migration, allow gas exchange between the wound and the environment, provide protection against bacterial infection, and be sterile, non-toxic, and non-allergenic [3]. The biomaterial that can be used as wound dressing for diabetic ulcers is a hydrogel. Hydrogel is a network polymer material that has a polymer chain that is very hydrophilic so that it can associate with large amounts of water without being dissolved [4]. Water can be bound to the polymer network or free to move within the polymer network. The moisture content in hydrogel can mimic biological tissue (about 70–99%) [5]. Due to its high-water content, hydrogel is biocompatible, making it a good candidate for diabetic ulcer wound dressing [6]. In addition, hydrogels have several desirable ideal characteristics, such as high adsorption capacity and good durability and stability in the working environment [7].

Silk fibers, protein-based biopolymers, are employed as a potential hydrogel for wound healing scaffolds [8]. Silk obtained from Bombyx mori, a domesticated silkworm species, is the most exploited [9]. The silk contains two main proteins, fibroin [9] and sericin. Silk sericin from the Bombyx mori silk cocoons has received much attention due to its outstanding properties. Sericin exhibits several promising biological activities such as antioxidant properties, the promotion of collagen formation, anti-inflammatory, and antimicrobial activity, and can be used in drug release biomaterials to promote stability and prolonged drug release [10]. Until now there have been several studies conducted on sericin-based hydrogels, such as sericin-poly(ethylene glycol) diacrylate hydrogels for berberine drug release [11], sericin-alginate hydrogels for drug release [12], and self-drinking sericin hydrogel assemblies [13]. It was found that sericin hydrogels have good swelling properties, high porosity, pH-responsive degradation, good adhesion and cell compatibility, and sustained drug release ability [13]. Kundu et al. [14] showed that a sericin-polyacrylamide hydrogel can be used as injectable hydrogel to increase tissue regeneration and cell scaffolding, especially for fibroblast cells. Sericin is also interesting to study because it can have a large economic, social, and environmental impact especially in countries that practice sericulture [10]. 

This work aimed to study the manufacture and potential of sericin protein-based hydrogels obtained from silkworm cocoons, with the addition of polyvinyl alcohol (PVA) to increase the stability of the resulting hydrogels. This research focuses on the characteristics of sericin and sericin/PVA hydrogels, such as pore morphology, functional groups, chemical structure, and degradation properties.

## 2. Results and Discussion

### 2.1. Isolation of Sericin

**Figure 1:**
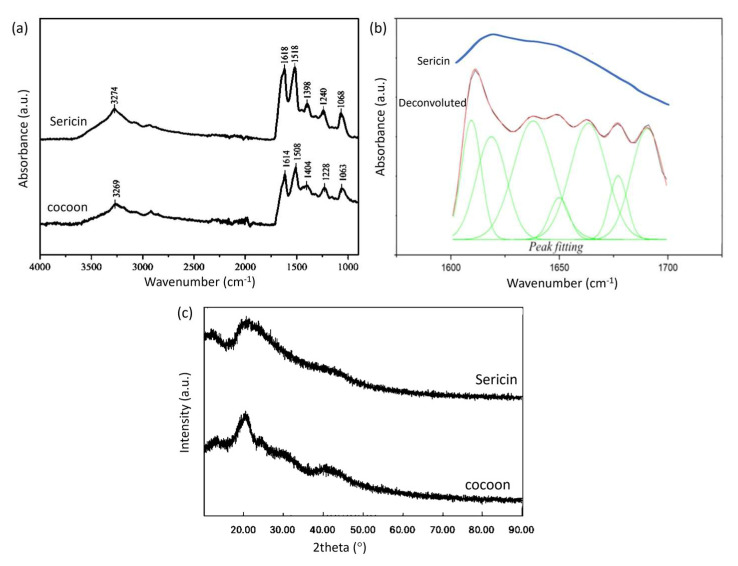
(**a**) FTIR spectra of cocoon and sericin (**b**) Fourier deconvolution results and Gauss peak fitting from the absorption of the amide group I of the FTIR spectrum of sericin (**c**) XRD patterns of cocoon and sericin.

The FTIR spectra of the cocoon and sericin can be seen in Figure 1a,b. There are three characteristic absorptions in the cocoon and sericin due to the presence of protein, namely absorption of the amide I group (1600–1700 cm^−1^), amide II group (1504–1582 cm^−1^), and amide III group (1200–1300 cm^−1^). Table 1 shows an analysis of the FTIR absorption peaks of the cocoon and sericin.

Zhang et al. [15] showed that the FTIR spectra of the sericin and cocoon are not much different, and fibroin has a strong peak around wavenumber 1450 cm^−1^. In contrast, sericin does not have a strong peak in this area. If we compare the FTIR spectra of sericin and cocoons, there is a peak shift, but the FTIR spectra of sericin and cocoons are not much different.

Proteins have a secondary structure, which is stabilized by hydrogen bonds in the polypeptide backbone [16]. Sericin has more than one secondary structure with overlapping absorption in the amide I absorption area that appeared as one absorption peak. A Fourier deconvolution was carried out on the absorption band of the amide I group to perform peak fitting [17,18]. The results of deconvolution, peak fitting, and the original spectrum from the absorption band of the amide group I of the sericin FTIR spectrum can be seen in Figure 1b.

After peak fitting, the position of each peak showed a specific secondary structure according to the literature. The absorption of β-sheet structures can be seen in the range 1610–1642 cm^−1^, as well as random coil in 1643–1650 cm^−1^, and β-turn in 1660–1669 cm^−1^ [19]. The area of each peak shows the proportion of the secondary structure in the sample. It was found that most of the sericin samples had a β-sheet secondary structure, as can be seen in Table 2. The sericin sample comes from a sericin solution which is frozen and then freeze-dried. In the freeze-drying process, the evaporated water causes the sericin polymer chains to aggregate to form a β-sheet structure [20]. The β-sheet structure decomposes when heated in water to form a water-soluble random coil structure. In the process of cooling and freeze–thawing, the random coil structure rearranges into a β-sheet again so that the solution can form a gel.

XRD results for the cocoon and sericin can be seen in Figure 1c. The cocoon shows an XRD peak at a diffraction angle of 20.5°, and sericin shows a peak at a diffraction angle of 21°. The XRD peak for the cocoon has a sharper shape. In contrast, the XRD peak for sericin has a broad and sloping peak shape, indicating that the cocoon and sericin have different crystallinities. The cocoon tends to be more crystalline. The difference in peak shape may be due to the extraction of sericin which changes its crystallinity or the presence of crystalline fibroin in the cocoon.

### 2.2. Preparation and Characterization of Hydrogels

The sericin-based hydrogel was synthesized using the freeze–thawing process to form hydrogels through physical crosslinks, forming polymer networks with non-covalent interactions [4]. In this method, a crosslinking agent does not need to be added [21]. We avoided the use of a chemical crosslinker for hydrogel stabilization. Unreacted cross-linker can be toxic to cells. Physical crosslink using the freeze–thawing method can enhance the stability of hydrogels without chemical residues or toxic compounds.

The separation of phases into a PVA-rich and water-rich phase is an essential mechanism during the gelation of freeze–thaw PVA hydrogels. During freezing, water freezes and expels PVA to create high PVA concentration areas, causing the phase separation of water and PVA. The crystallite and hydrogel bonds started forming during the freezing or nucleation of water molecules within the PVA solution since the PVA chains got closer to each other. Following thawing, the contacts remain, creating a non-degradable three-dimensional hydrogel network. Phase separation helps PVA crystals to maintain the crystallite region even after thawing [22]. In the thawing process, ice crystals melt to form pore structures. As the number of freeze–thawing cycles increases, the polymer chains can move closer to each other, thereby facilitating the formation of hydrogen bonds and crystallization. 

More freezing-thawing cycles will form more hydrogen bonds, resulting in a more stable and rigid hydrogel. The crystalline regions formed to contribute to the strength properties of the hydrogels. The higher the number of cycles, the better the stability, mechanical strength, and elasticity of the resulting hydrogels [23,24]. The addition of PVA to the sericin hydrogel was carried out to improve the stability and mechanical properties of the resulting hydrogel because PVA hydrogel has a high modulus of elasticity and mechanical strength [25].

The separation phase also occurs in sericin/PVA systems, resulting in a crosslink between these two polymers. However, the number of functional groups that can form crosslinks is much smaller in sericin–sericin and sericin–PVA than in PVA–PVA. Therefore, the bonds formed are relatively weaker, causing less stable three-dimensional bonds for sericin–PVA. 

**Figure 2:**
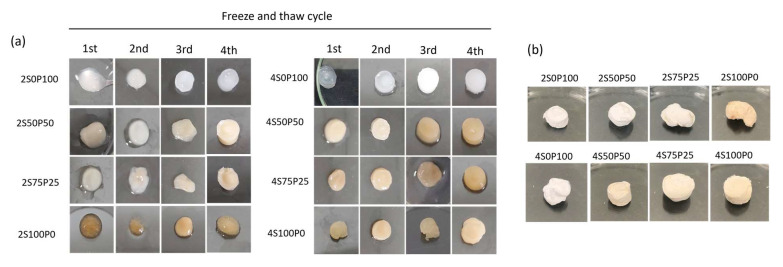
(**a**) Composition and freeze–thawing cycle effect on the physical appearance of the hydrogel (**b**) freeze-dried hydrogel after four cycles of freeze–thawing. S and P denote sericin and PVA with various volume ratio.

Figure 2a shows the hydrogels formed by various freeze–thawing cycles with 2% and 4% polymer concentrations. It shows that the addition of PVA can increase the stability of the hydrogel. Pure sericin hydrogels (2S100P0 and 4S100P0) tend to form hydrogels that are less able to maintain their shape and are quite brittle. The higher the ratio between the PVA solution and the sericin solution in the hydrogel, the more stable the hydrogel produced. These results are in agreement with the work reported by Tao et al. [26]. The difference in molecular weight of the sericin and PVA polymer chains might influence the hydrogel’s stability. The PVA has a high molecular weight and degree of hydrolysis. On the other hand, sericin may contain lower molecular weight polymer chains, contributing to the hydrogel’s lower stability. These factors will play a role in intermolecular and intramolecular hydrogen bonding and crystallinity [23].

Meanwhile, the sericin/PVA mixture with a concentration of 4% produced a more stable hydrogel. The hydrogels that seemed the most stable were the 4S50P50 and 4S0P100 hydrogels. Hydrogels containing sericin tended to be yellowish, while the hydrogel results from previous studies were white [26]. The impurity of the sericin may cause this yellowish color because the extraction process does not involve a purification process.

Figure 2b shows the freeze-dried hydrogels after four cycles of the freeze–thawing process. We observed that hydrogels with a higher ratio of sericin content tend to have brittle properties, while hydrogels with a higher ratio of PVA content have sponge-like properties.

Scanning Electron Microscopy (SEM) was carried out on all hydrogel samples to obtain surface morphology and cross-sectional data. The purpose of SEM was to analyze the pore size and distribution of the resulting hydrogels. The porosity of a hydrogel is an important and necessary thing in applying hydrogels as wound dressings or scaffolds. Hydrogels with good porosity (pore size and distribution) and interconnected pores will have good transport routes for water, drugs, and nutrients because the substance in the hydrogel uses the hydrogel pores as a pathway to enter and leave the hydrogel. In addition, the presence of pores in the hydrogel is expected to increase oxygen permeation in the area around the wound so that the wound environment remains moist and accelerates the healing process [27].

**Figure 3:**
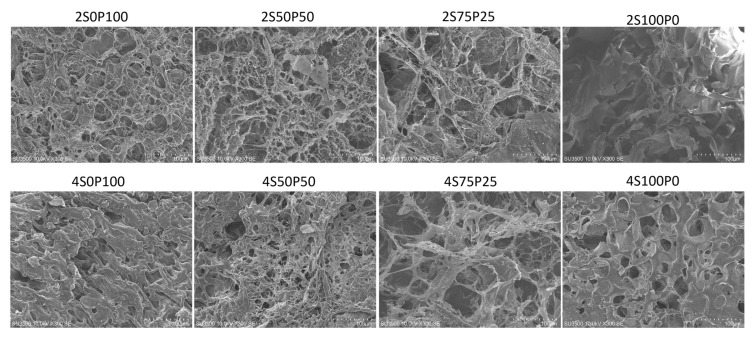
SEM images of the hydrogel surface at a concentration of 2% and 4% with various sericin and PVA ratio compositions.

Figure 3 presents SEM images of the surface morphology of hydrogel samples with PVA and sericin concentrations of 2% and 4%. There is a reasonably good pore distribution in the hydrogel samples containing PVA and sericin/PVA at concentrations of PVA and sericin 2%, with larger pore sizes as the volume ratio of sericin used increases. The pore size of the 2S75P25 sample is larger than the 2S50P50 and the 2S0P100. It can also be seen in Figure 3 that there is a significant difference in the surface morphology of sample 2S100P0 (100% sericin) when compared to other samples. The 2S100P0 sample shows a sheet-like structure, while the three other samples have a net-like structure. The morphological differences were due to the lack of ability of the 100% sericin hydrogel system to form crosslinks during the freeze–thawing process. The low number of crosslinks formed in the 100% sericin (2S100P0) hydrogel resulted in the formation of a net structure that was sufficiently stable so that the result was a hydrogel with poor structural stability (as shown in Figure 3) and a weak ability to trap water and other substances.

The hydrogel samples with 4% PVA and sericin concentrations had different pore sizes and walls compared to hydrogel systems with equivalent volume ratios of PVA and sericin at 2% concentration. The relationship between the formation of hydrogel crosslinks and the concentration of the hydrogel constituent solution can cause this difference. The higher the concentration of sericin solution used, the better the crosslinks in the hydrogel will form. Therefore, generally, a hydrogel with a concentration of 4% has thicker walls than one with 2%.

**Figure 4:**
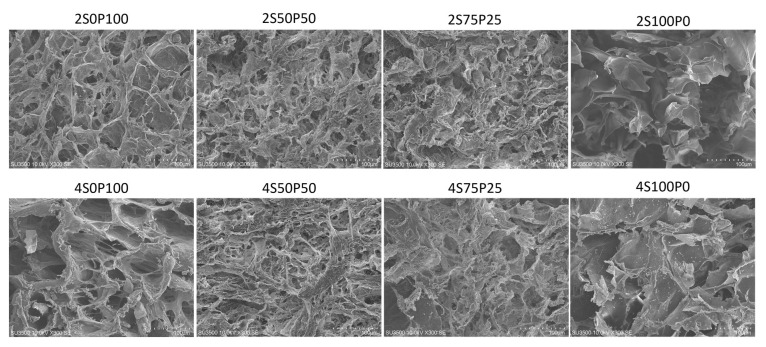
SEM images of the hydrogel cross-section at a concentration of 2% and 4% with various sericin and PVA ratio compositions.

SEM characterization was also carried out on the cross-sectional surface of the hydrogel samples. The cross-sectional area shows the internal morphology of the hydrogel sample with interconnected pores, as shown in Figure 4 below:

All hydrogels with PVA and sericin concentrations of 2% and 4% had porosity inside the hydrogel structure. The porosity in that section reflects good porosity in the internal part of the hydrogel. It was also seen that the 2S0P100, 2S50P50, and 2S75P25 hydrogels had porosity in the surface and cross sections. The same trend was also found in samples with a concentration of 4%. It can be concluded that all hydrogels with good porosity throughout the hydrogel were formed using the freeze–thawing method.

The 2S100P0 sample also showed better pores and walls in the hydrogel structure compared to the 2S100P0 sample in the SEM characterization of the surface area. However, overall, the results of the images in the sectioned area show that the 2S100P0 sample still has quite poor crosslinked structures and hydrogel stability. The low number of crosslinks between sericin polymer chains may be responsible for the hydrogel instability.

Based on Table 3 and Table 4, the pore diameter of the sericin/PVA hydrogel has an average size of about 44–97 µm. The pore diameter indicates that the resulting sericin/PVA hydrogel system can function as a promising wound scaffold for skin tissue (such as for skin regeneration) which generally has an optimum pore diameter size of 20–125 µm [28].

**Figure 5:**
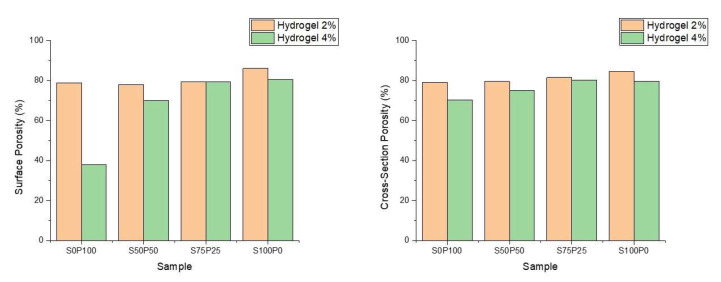
The porosity of the sericin/PVA hydrogels resulting from SEM image analysis on the sample surface and cross-section. Hydrogels 2% and 4% denote the concentration of sericin and PVA.

Furthermore, through data analysis performed using the ImageJ application, the porosity of each hydrogel was obtained on the surface and cross-sectional sections of the hydrogel. The two graphs in Figure 5 show that all sericin/PVA hydrogels have about 70–85% porosity. Thus, all PVA–sericin hydrogel samples have good enough porosity to function as wound scaffolds which generally have an ideal porosity of around 60–90% [29].

Figure 5 shows that the hydrogel with a concentration of 2% PVA and sericin has a higher porosity than the hydrogel with a similar composition of PVA and sericin at a concentration of 4%. The 2% PVA hydrogel structure has thinner walls of the and the formation of fewer crosslinks in the hydrogel. The difference in the porosity of the hydrogels at various concentrations was insignificant, considering that the difference between the porosity of the sericin/PVA hydrogels at concentrations of 2% and 4% was not more than 10%. Thus, the sericin/PVA hydrogels produced had high porosity and were suitable for wound dressing.

### 2.3. Degradation Test

A degradation test using PBS was conducted to simulate how the hydrogel would behave in the body, when used as a scaffold and wound dressing. The hydrogels were not intended to dissolve, yet we immersed all samples in a PBS solution. A degradation test was carried out on all sericin/PVA hydrogels (concentrations of 2% and 4%) in a pH 7.4 PBS solution to simulate hydrogel degradation. The degradation test was carried out for 672 h or 28 days at a room temperature of around 25 °C. Degradation performance is crucial in applying material as a scaffold and wound dressing. A scaffold or wound dressing needs to have a slow degradation rate to maintain its structure and function in cell and tissue regeneration for a specific period [30]. PBS solution was selected as the degradation medium to provide the closest condition for interaction between the hydrogel and skin in the biological environment [25].

**Figure 6:**
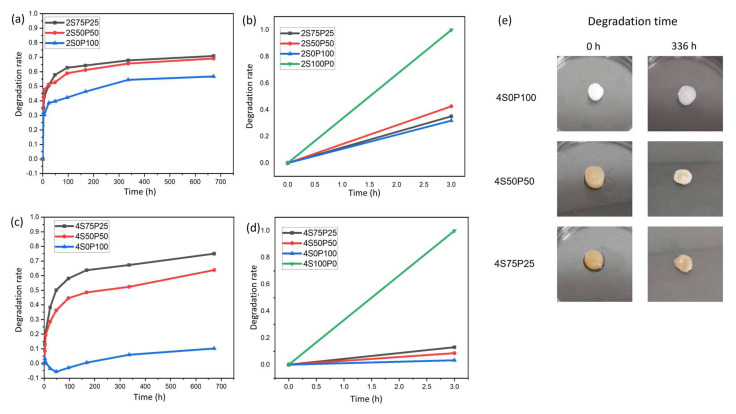
Hydrogel degradation rate after 672 h and initial 3 h for samples with a concentration of (**a**,**b**) 2% and (**c**,**d**) 4%. (**e**) Visual data of 4% hydrogel sample before and after 336 h degradation test.

The results of the degradation test in the first 3 h showed that the hydrogel with 100% sericin composition (2S100P0 and 4S100P0) had undergone complete degradation in PBS solution before reaching the 3 h time. The degradation rate is very high, especially compared to the degradation rates of other hydrogel samples, as shown in Figure 6b,d. In this case, the degradation of sericin results from dissolution in PBS. Crosslinks formed from hydrogel bonds between sericin chains cannot provide stability against the swelling and dissolution processes in PBS.

The addition of PVA decreased the rate of degradation of the sericin hydrogel. Crosslinks formed between sericin and PVA so that the network structure in the hydrogel became difficult to break by the PBS solution. The stability effect of the PVA hydrogel is indicated by the decrease in degradation rate in samples containing PVA, compared to 100% sericin (2S100P0 and 4S100P0). The sericin/PVA hydrogel did not experience total degradation for up to 672 h. The degradation rate of PVA–sericin itself is directly affected by the amount of PVA added to the hydrogel system. The higher the amount of PVA in a sericin/PVA hydrogel, the lower the hydrogel degradation rate. We observed the degradation rate of the S7525 series hydrogel, which has a PVA content of 25% higher (70–75%) than the S50P50 series hydrogel with a PVA content of 50%, which was around 63–69% after 672 h. All PVA–sericin hydrogel samples had a degradation rate indicating the hydrogel’s potential as a wound scaffold, especially for skin tissue regeneration, which has a degradation time of around 3 to 4 weeks [31]. Even though sericin will experience dissolution when applied as a wound dressing, this will not be a problem. The released sericin is expected to help improve the wound-healing process and induce cell growth [32]. Meanwhile, the porous hydrogel can become a place for cells to attach and grow.

In addition to the amount of PVA content in the sericin/PVA hydrogel system, the concentration of PVA and sericin also affected the stability of the resulting hydrogels. Hydrogels with a concentration of 2% had a higher degradation rate than 4%. The difference in degradation rate was due to the hydrogel structure of the 2% PVA and sericin concentration which had higher porosity and thinner walls. The hydrogel structure at PVA and 2% sericin concentrations caused the hydrogel to be more susceptible to structural damage during hydrogel immersion in PBS solution. The structure of the hydrogel itself is also the main factor causing the 100% pure sericin hydrogel system to degrade very quickly. As seen in the surface and cross-section SEM results of the S100P0 series hydrogel, the hydrogel with 100% sericin composition has a very open hydrogel structure with very few crosslinked walls. Thus, in this degradation test, the S100P0 series hydrogel very quickly experienced degradation during immersion in PBS solution.

There were also phenomena other than hydrogel sample degradation that occurred during this degradation test, especially in the 4S0P100 sample. The 4S0P100 sample experienced a negative degradation rate for some time, indicating a swelling phenomenon. It resulted in an increase in hydrogel mass. The swelling phenomenon can also be seen through qualitative observations in the form of taking visual hydrogel data after immersion for a certain period.

The hydrogel visual data in Figure 6e shows that due to the low degradation rate and the occurrence of swelling in the 4S0P100 sample, there was no significant change in the structure, shape, and size of the hydrogel even after 672 h of degradation testing. Meanwhile, the 4S50P50 and 4S75P25 samples showed significant changes in the structure, shape, and size of the hydrogels, indicating degradation. Gradual changes occurred in some samples, which made the samples smaller, thinner, and lost the water content in them, so they could not properly maintain the stability of the hydrogel structure and shrank.

**Figure 7:**
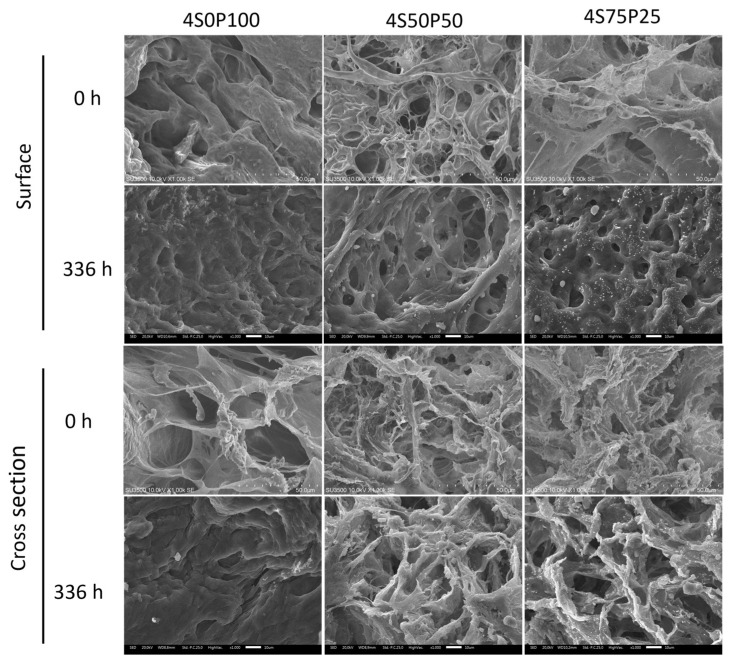
Surface (**top**) and cross-section (**bottom**) morphology of 4% PVA-sericin hydrogel before PBS solution immersion and after 336 h (14 days) PBS solution immersion.

It was also seen that there was a color change in the hydrogel containing sericin, indicating that during the degradation and immersion process, sericin dissolved in the PBS solution and was degraded from the crosslinked network in the hydrogel. SEM images of samples 4S0P100, 4S50P50, and 4S75P25, as shown in Figure 7, show that the degradation process of the sericin/PVA hydrogel in PBS solution causes damage to the secondary bonds that make up the hydrogel. The breaking of the crosslink bonds in the hydrogel leads to a change in the structure and shape. The formation of space causes new pores in the hydrogel structure after the polymer chains. Furthermore, the hydrogel is damaged or separates due to the degradation. There is a thickening of the walls of the hydrogel structure after immersion in the PBS solution. The addition to the hydrogel wall thickness was caused by swelling of the PVA chains in the PVA–sericin hydrogel.

## 3. Conclusions

The autoclaving method successfully extracted sericin from silkworm cocoons. Hydrogels of 2% and 4% sericin/PVA with ratios of 4:0, 3:1, 2:2, and 0:4 (*v*/*v*) were successfully prepared using the freeze–thawing method. Sericin/PVA hydrogel stability is affected by its composition, in which the 4% concentration is more stable than 2%. From the results of FTIR and XRD characterization, the 4% hydrogel with a ratio of 1:1 PVA and sericin has characteristics that are a combination of pure PVA and pure sericin. All hydrogels had an average pore size capable of meeting the skin regeneration requirements and sufficient porosity to meet the ideal scaffold porosity. The sericin/PVA hydrogel system with a concentration of 2% and 4% had good porosity (70–85%). The rate of degradation of the PVA–sericin hydrogel system after 672 h demonstrated the potential of the PVA–sericin hydrogel as a wound scaffold, particularly for skin tissue regeneration.

## 4. Materials and Methods

### 4.1. Materials

Cocoons were obtained from the local market and used as a source of sericin. PVA fully hydrolyzed 99% with Mw 146,000–186,000 mol/g (Sigma Aldrich, St. Louis, MI, USA) and PBS pH 7.4 analytical grade were used without further purification.

### 4.2. Isolation of Sericin

Sericin was isolated using an autoclave method. The cocoons were cut into small pieces, then washed thoroughly with demineralized water to remove the remaining dirt and dried for 2–3 days at room temperature. The cocoon was mixed with demineralized water with a ratio of 1:30 and then heated at 121 °C in an autoclave for 30 min to dissolve the sericin. The sample was cooled down to room temperature. The sample was then filtered using filter paper. The resulting filtrate containing sericin was then frozen at −20 °C and lyophilized (Freezone 4.5 L–Labconco) to obtain a white sericin powder. The sericin powder was ground using a mortar to obtain a fine and homogenous powder.

### 4.3. Preparation of Sericin/PVA Hydrogels

Sericin/PVA hydrogels were prepared using the freeze–thawing method. The variations of each PVA, sericin, and sericin/PVA hydrogel composition are shown in Table 5. PVA solution was prepared by incorporating PVA powder into demineralized water, then heating and stirring it using a hot plate at 85 °C to obtain a PVA solution with concentrations of 2% and 4% (*w*/*v*). Sericin solution was prepared by incorporating sericin powder into demineralized water and then heating it at 85 °C to obtain a sericin solution with concentrations of 2% and 4% (*w*/*v*). Then, the PVA solution and sericin solution were mixed at 85 °C for 20 min with a volume ratio of sericin to PVA of 4:0, 3:1, 2:2 and 4:0, as listed in Table 5. The sericin/PVA solution was poured into a 24-well plate. The solution was frozen at −20 °C for 20 h to induce crystallization (freezing), then allowed to stand at room temperature (thawing) for 4 h. The freeze–thawing cycle was carried out 4 times. The resulting hydrogel sample was then freeze-dried for 24 h to obtain a porous sericin/PVA hydrogel. Samples containing 100% PVA (2S0P100 and 4S0P100) were used as control.

### 4.4. Characterizations

#### 4.4.1. FTIR-ATR Spectroscopy

Functional group analysis of the hydrogels was carried out using the FTIR-ATR spectrometry method (Alpha II Bruker) with diamond crystals. The detector used has a wave number of 500–4000 cm^−1^ with a resolution of 2.0 and a total of 50 scans. A sample shuttle measurement was performed to interleave sample and background scan.

The absorption peak of sericin was analyzed using Fourier deconvolution in the absorption band area of the amide I group (1600–1700 cm^−1^) [17,18]. The secondary structure was calculated by this method to determine the percentage of sericin with β-sheet, random coil, and β-turn structures.

#### 4.4.2. XRD Characterization

The sericin structure was identified using XRD analysis, measured on the D8 Advanced Eco Bruker tool using a Cu anode with a wavelength of 1.5406 Å. The data were collected over the 2θ range of 10–90°.

#### 4.4.3. Morphological Characterization

SEM characterization was carried out for freeze-dried hydrogel specimens using a scanning electron microscope (SEM SU3500). The specimen was coated with gold to make it conductive using a Hitachi MC1000 Sputter at an accelerated voltage of 10.0 kV.

Morphology images were obtained from both surface and cross-section SEM images of each sample variation. The porosity analysis was conducted by increasing the image contrast by 25% to obtain clearer color differences and minimize porosity analysis errors due to the presence of pores with orientations that were not perpendicular to the SEM image which causes these pores to not be seen clearly. Then, the porosity value was obtained using the particle analysis feature in ImageJ. To obtain quantitative data, pore size analysis was conducted using ImageJ by measuring the diameter of random pores from the SEM image.

### 4.5. Degradation Test

The PVA–sericin hydrogel samples were tested for their degradation rate by periodically measuring the wet mass after the hydrogel was placed in the desired simulated conditions. The initial mass of the hydrogel was measured first. Degradation studies were carried out for 28 days via continuous immersion of the samples in PBS solution. Hydrogel samples had a diameter of about 0.8–1.7 cm and a height of about 0.3–0.5 cm. The hydrogel (*n* = 5 per time point) was placed in a 5 mL PBS solution pH 7.4 at room temperature to simulate the acidity level of the hydrogel working environment. Then, the wet mass of the sample was measured with the timepoints of 6 h, 12 h, 1 day, 2 days, 3 days, 4 days, 7 days, 14 days, and 28 days. The mass change data that has been obtained was then processed to obtain the sample degradation rate for each specific time using the equation:

(1) $$Degradation\;ratio\;\left(w/w\right)=\frac{\left(Wo-Wi\right)}{Wo}$$

where *Wo* is initial weight of hydrogel and *Wi* denotes the final wet mass of hydrogel after degradation. After the degradation test was completed, SEM measurements were carried out on samples that had undergone degradation. Samples after 336 h immersed in PBS were taken out and freeze-dried for 24 h to obtain dried hydrogels. Surface and cross-section area of the degraded hydrogels were characterized using SEM. Untreated hydrogels were used as control.

## Figures and Tables

**Table 1:** FTIR absorption peaks of cocoon and sericin.

Absorption Peak (cm^−1^)		Functional Groups
Cocoon	Sericin	
3269	3274	N-H stretching overlapping with OH from serine (3500–3200 cm^−1^)
1614	1618	Amide I group mostly represents the C=O stretching of the amide group
1508	1518	Amide II group, contribution from N–H bending and C–N stretching
1404	1398	C–H and O–H bending
1228	1240	Amide III group, C–N and C=O stretching
1063	1068	C–OH stretching

**Table 2:** Secondary structure of sericin.

Secondary Structure	Percentage (%)
β-sheet intermolecular	27.63
β-*sheet*	24.18
*Random coil*	4.18
β-*turn*	44.01

**Table 3:** The average pore diameter on the surface of the hydrogel samples.

Sample	Diameter (µm)		
	Minimum	Maximum	Mean
2S0P100	24	110	60
2S50P50	30	118	59
2S75P25	32	138	75
2S100P0	-	-	-
4S0P100	27	69	44
4S50P50	27	71	48
4S75P25	39	123	81
4S100P0	33	139	71

**Table 4:** The average pore diameter in the cross-section of the hydrogel samples.

Sample	Diameter (µm)		
	Minimum	Maximum	Mean
2S0P100	34	123	67
2S50P50	37	85	56
2S75P25	33	99	65
2S100P0	-	-	-
4S0P100	43	140	72
4S50P50	38	67	51
4S75P25	52	112	69
4S100P0	35	191	97

**Table 5:** Hydrogel compositions.

Polymer Concentration	Sample	Sericin to PVA Ratio (*v*/*v*)
2%	2S100P0	4:0
	2S75P25	3:1
	2S50P50	2:2
	2S0P100	0:4
4%	4S100P0	4:0
	4S75P25	3:1
	4S50P50	2:2
	4S0P100	0:4

## Data Availability

Not applicable.
